# X Chromosome-Linked CNVs in Male Infertility: Discovery of Overall Duplication Load and Recurrent, Patient-Specific Gains with Potential Clinical Relevance

**DOI:** 10.1371/journal.pone.0097746

**Published:** 2014-06-10

**Authors:** Chiara Chianese, Adam C. Gunning, Claudia Giachini, Fabrice Daguin, Giancarlo Balercia, Elisabet Ars, Deborah Lo Giacco, Eduard Ruiz-Castañé, Gianni Forti, Csilla Krausz

**Affiliations:** 1 Department of Experimental and Clinical Biomedical Sciences, University of Florence and Centre of Excellence DeNothe, Florence, Italy; 2 Division of Endocrinology, Department of Clinical and Molecular Sciences, Umberto I Hospital, Polytechnic University of Marche, Ancona, Italy; 3 Molecular Biology Laboratory, Fundació Puigvert, Universitat Autonoma de Barcelona, Barcelona, Spain; 4 Andrology Service, Fundació Puigvert, Universitat Autonoma de Barcelona, Barcelona, Spain; Clermont-Ferrand Univ., France

## Abstract

**Introduction:**

Spermatogenesis is a highly complex process involving several thousand genes, only a minority of which have been studied in infertile men. In a previous study, we identified a number of Copy Number Variants (CNVs) by high-resolution array-Comparative Genomic Hybridization (a-CGH) analysis of the X chromosome, including 16 patient-specific X chromosome-linked gains. Of these, five gains (DUP1A, DUP5, DUP20, DUP26 and DUP40) were selected for further analysis to evaluate their clinical significance.

**Materials and Methods:**

The copy number state of the five selected loci was analyzed by quantitative-PCR on a total of 276 idiopathic infertile patients and 327 controls in a conventional case-control setting (199 subjects belonged to the previous a-CGH study). For one interesting locus (intersecting DUP1A) additional 338 subjects were analyzed.

**Results and Discussion:**

All gains were confirmed as patient-specific and the difference in duplication load between patients and controls is significant *(p = 1.65×10^−4^).* Two of the CNVs are private variants, whereas 3 are found recurrently in patients and none of the controls. These CNVs include, or are in close proximity to, genes with testis-specific expression. DUP1A, mapping to the PAR1, is found at the highest frequency (1.4%) that was significantly different from controls (0%) (*p = 0.047* after Bonferroni correction). Two mechanisms are proposed by which DUP1A may cause spermatogenic failure: i) by affecting the correct regulation of a gene with potential role in spermatogenesis; ii) by disturbing recombination between PAR1 regions during meiosis. This study allowed the identification of novel spermatogenesis candidate genes linked to the 5 CNVs and the discovery of the first recurrent, X-linked gain with potential clinical relevance.

## Introduction

Infertility is a multi-factorial disorder affecting approximately 15% of couples – half of these can be attributed to the male. Currently known causes of male-factor infertility account for only 60% of cases and known genetic factors contribute to about 15% of severe male factor infertility [1]. The most frequent molecular genetic cause is related to the Y chromosome and concerns the AZF deletions [2]. These deletions are the first example in andrology of functionally-relevant CNVs and can be easily studied with plus/minus PCR. Recently, the development of high-throughput analytical techniques such as a-CGH have allowed the screening of large numbers of loci and have been used with the principal aim of identifying novel spermatogenesis candidate genes. These studies have also been useful in identifying a CNV burden in infertile men, mainly involving the sex chromosomes [3]–[5].

Considering the high complexity of spermatogenesis, which requires more than 2,000 genes, it is highly likely that a proportion of the 40% ‘missing’ aetiology is linked to yet unknown genetic factors [1]. CNVs may induce a pathogenic effect in a number of ways: structural changes to regulatory regions or a numerical increase or decrease in protein-coding regions may have a direct effect on mRNA levels [6]; large-scale CNVs may cause changes to the well-regulated 3D structure formed by chromatin [7], leading to downstream effects on the regulation of protein-coding regions. Finally, large CNVs may also disturb chromosome pairing at the PAR regions during meiosis [8], [9].

While the AZF region-linked genes have been extensively studied in respect to male infertility [10] very few studies have focussed on the X chromosome, despite its predicted enrichment in genes expressed in the testis [11], [12]. Only a single X-linked gene has been shown to definitively contribute to an infertility phenotype, the androgen receptor (*AR*) [1], leaving huge scope for further experiments on this chromosome. Recently, evolutionary models demonstrated that the X chromosome progressively accumulating testis-expressed genes [11]. Accordingly, 18% of the 800 protein-coding genes on the human X chromosome do not show orthologs in other placental mammals. Of these, the majority have been acquired by the human X chromosome *de novo* or through transposition from the autosomes. Two-thirds of these are ampliconic, possessing duplicated 10 Kb segments with >99% homology and are predicted to be involved in male fitness [12].

In our previous study [3], we analyzed the CNV status of 96 infertile patients and 103 controls using a custom-designed 8×60 K microarray targeting the X chromosome (Agilent Technologies, Santa Clara, CA, USA). Of the 44 gains identified, 16 were patient-specific and the five most promising CNVs (DUP1A, DUP5, DUP20, DUP26 and DUP40) were selected for testing in an enlarged study population.

## Materials and Methods

The local Ethics Committees of the University Hospital Careggi and the Fundació Puigvert approved the study and consent procedure. All participants gave written, informed consent. The consent forms are stored locally at the University Hospital Careggi. All data were analysed anonymously.

### CNV Selection and Bioinformatic Analysis

Five CNVs (DUP1A, DUP5, DUP20, DUP26 and DUP40) were selected from the 16 patient-specific gains identified in our previous study [3]. CNVs underwent several selection steps. Initially, this focused on the frequency at which CNVs were identified in the a-CGH study. CNVs found in control samples were excluded. Online data-sources, such as OMIM, gene ontology terms, and literature review were used to find candidate features within 0.5 Mb of the CNV minimum. Information about expression data was obtained from microarray and RNA-seq experiments deposited in the GermOnline database [13], [14]. CNVs containing genomic features with a potential involvement in spermatogenesis were selected. At this regard, DUP1A resulted of particular interest and therefore it was subjected to a deepened investigation. All genomic *loci*, in the previous study reported in Hg18 genome assembly [3], have been converted to Hg19 assembly and all loci in the current study are reported according to Hg19.

### Study Population

Patients with non-Italian or non-Spanish origin were excluded. Patients were recruited after a comprehensive clinical examination in both centres using the same clinical protocol, according to WHO guidelines [15]. Exclusion criteria included all known causes of infertility: history of mono- or bilateral cryptorchidism, varicocele (grades 2 and 3), obstructive azoospermia, recurrent infections, iatrogenic infertility, hypogonadotrophic hypogonadism, karyotype anomaly and Y-chromosome microdeletions. Control samples were recruited from pre-vasectomy patients with proven fertility, male partners of infertile women (tubal factor, anovulation and endometriosis) and male volunteers from the general population. Given that the aim of the study was to define the effect of CNVs on spermatogenesis, the control group contained only subjects presenting with normal sperm parameters (according to the WHO manual). Additional information about sperm parameters is provided in the supplementary material and methods S1 in File S1.

In addition to the previous study population of 199 samples (96 patients and 103 controls) [3], 404 new subjects (180 patients and 224 controls) were analyzed at the 5 selected loci. The total patient group (n = 276) used in this study consisted of 96 azoospermic, 83 cryptozoospermic (<1.0×10^6^ spermatozoa. ml^−1^) and 97 severe oligozoospermic subjects (between 1.0×10^6^ and 5.0×10^6^ spermatozoa. ml^−1^) of which 130 were Spanish and 146 Italian. The control group (n = 327) consisted of 107 Spanish and 220 Italian normozoospermic subjects. For DUP1A only, the further enlargement of the study population with additional 158 patients (27 azoospermic, 59 cryptozoospermic and 72 severe oligozoospermic) and 180 controls ensured the analysis of a total of 941 subjects.

### qPCR Analysis

The copy number state of each locus was determined by qPCR. Primer sequences are reported in Table S2 in File S1. Before testing, all samples were put through rigorous quality control to ensure that DNA quality and concentration was sufficient. DNA samples that showed contamination were re-precipitated, using ethanol precipitation. DNA samples were diluted to 50 ng.µL^−1^ in ddH_2_0. Each sample was analyzed in triplicate in a 96-well plate. The SYBR Select Master Mix produced by Invitrogen (REF: 4472908) was used. For each sample, *PMP22* was amplified as a reference gene for analysis purposes. The reaction conditions were as follows: 20 ng DNA; 200 nM Primer (forward and reverse); SYBR Green SELECT Master mix (1x concentration) in a total reaction volume of 20 µL. In the case of DUP1A, an additional qPCR was performed in order to test the *LINC00685/PPP2R3B* gene dosage ratio. For this purpose, we tested the *PPP2R3B* copy number state in DUP1A carriers using a pair of primers solely mapping to the *PPP2R3B* gene. We used the same reaction conditions, but optimal concentration for these primers was 400 nM (forward and reverse). qPCR was performed on TaqMan 7900 HT on ‘Absolute Quantification’, using the pre-set ‘Standard’ cycle conditions. The annealing temperature was 60°C. A non-targeting control and a ‘duplicated’ control (a Klinefelter 47, XXY man) was included on each plate. For Dup1A, we simultaneously analysed sample 08–373 (carrying DUP4A) and A800 (carrying DUP1A) for whom CNVs had previously been identified by a-CGH (3). Threshold cycle and baseline were calculated automatically and relative quantification was determined using the ΔΔCt analysis method.

All samples from the original a-CGH experiment (96 patients and 103 controls) were re-tested using the qPCR method validating a-CGH results. A single ‘normal’ result (absence of duplication in the triplicate) was required to denote a normal gene copy number. In case duplication was found, the analysis was repeated once for confirmation. A ‘borderline’ range was also possible and samples within this range were repeated; if a borderline result turned into duplication, another experiment was performed for confirmation.

### Statistical Analysis

SPSS software (version 20.0, Chicago, IL, USA) was used. CNV frequencies were analysed for significance using Fisher’s Exact Test in the ‘R’ software package and corrected using Bonferroni-Holm step-down correction for multiple testing.

## Results

### Case-control Association Study

The 5 selected CNVs (DUP1A, DUP5, DUP20, DUP26 and DUP40) were confirmed as patient-specific. Among the 276 patients analyzed, DUP5 was found in two (0.72%), DUP20 and DUP26 in one (0.36%), DUP40 in 3 (1.09%) and DUP1A in 4 patients (1.44%). Given the higher frequency of DUP1A, additional samples were tested for this CNV. In this enlarged study population, we found DUP1A in a further two patients (2/158; 1.26%) and in none of the additional 180 controls. Considering the total study population used for DUP1A only (434 patients and 507 controls), the difference in duplication frequency between patients and controls for this CNV reached statistical significance after Bonferroni-Holms correction for multiple testing (6/434 patients versus 0/507 controls; p = 0.047) (Table 1). DUP1A carriers displayed a heterogeneous semen phenotype ranging from azoospermia to severe oligozoospermia (Table 2). A phenotypic description of carriers of all CNVs is shown in Table 2.

**Table 1:** CNV frequency and statistical analysis of case-control association study.

CNV	Frequency Patients	Frequency Controls	Raw p-value	Corrected p-value^1^
DUP1A	6/434 (1.38%)	0/507	0.01	0.047
DUP5	2/276 (0.72%)	0/327	0.21	0.63
DUP20	1/276 (0.36%)	0/327	0.45	0.45
DUP26	1/276 (0.36%)	0/327	0.45	0.45
DUP40	3/276 (1.09%)	0/327	0.097	0.39

1 *Corrected using Bonferroni-Holm Step-down correction for multiple testing.*

**Table 2:** Phenotypic Description of CNV carriers.

CNV	Carrier Code	Sperm Conc. (10^6^ Spzoa. mL^−1^)	Total Sperm Count (10^6^ Spzoa)	Total Motile Sperm Count (10^6^ Spzoa)	FSH (U.L^−1^)	LH (U.L^−1^)	Testosterone (nmol.L^−1^)	Mean testicular volume^3^ (mL)
DUP1A	05–002	1.40	2.1	0.48	11.6	5.34	24.2	11
	08–093	0.18	0.4	0.1	9.63	N^1^	N/A^2^	14
	08–280	1.83	8.5	0.9	10.2	3.71	21	15
	11–262	0.0	0.0	0.0	26.3	11.5	20.8	14
	13–099	4.00	16.0	1.28	5.94	3.58	17.5	18
	A800	0.50	2.0	0.36	4.91	3.8	16.2	14
DUP5	08–280	1.83	8.5	0.9	10.2	3.71	21	15
	A760	0.0	0.0	0.0	3.23	4.64	7.1	15
DUP20	M9	5.00	7.5	3.6	4.5	3.5	N^1^	12
DUP26	07–013	0.0	0.0	0.0	10	2.2	N/A^2^	18
DUP40	05–238	0.22	0.22	0.0	5.0	N^1^	N/A^2^	13.5
	07–002	0.0	0.0	0.0	4.1	2.8	15.6	15
	A238	0.01	0.01	0.0	6.5	4.3	N^1^	14

1 *Reported in the medical history as within the “normal range”.*

2 *Not Available, but according to medical history “No signs of hypoandrogenism”.*

3 *Testis volume was determined using the ‘Prader’ orchidometer;*

*Spzoa = Spermatozoa.*

A comparison of sperm parameters between all CNV carriers and non-carriers was performed and is shown in Table S1 in File S1. The most relevant results concern the Total Motile Sperm count, which resulted significantly lower in carriers of DUP1A (p = 0.008), DUP26 (p = 0.003) and DUP40 (p = 0.03) compared to non-carriers.

### Bioinformatic Analysis for Physical Characterization of CNVs

The physical characteristics of the selected CNVs are shown in Table 3. DUP1A and DUP5 were of special interest because of their large size and location on the PAR1. Locations of DUP20, DUP26 and DUP40 are Xp22.2, Xp21.1 and Xq21.1, respectively. No homology of sufficient size for non-allelic homologous recombination (NAHR) was found between the upper and lower boundaries of all CNVs indicating that a mechanism other than NAHR might have led to the formation of these recurrent gains. We checked for other types of repeated elements that could contribute to genome instability, such as *Alu* elements, and found that, for DUP1A, DUP5, DUP20 and DUP26, the flanking regions are filled with short interspersed nuclear elements (SINEs), which include *Alus*, and long interspersed nuclear elements (LINEs). As for DUP40, two LINE elements, belonging to the L1PA3 family1, are located at the extremities of the CNV. Hence, we propose that the presence of SINEs and LINEs might underlie the generation of recurrent duplications.

**Table 3:** Physical Characteristics of CNVs selected for the study.

CNV	Start-end position(CNV Min)^1^	Size(Min)	Size(Max)	Substratefor NAHR^2^	Protein Coding within, ornearby CNV minimum(within 0.5 Mb)	Regulatory/RNA within, or nearby CNVminimum (within 0.5 Mb)
DUP1A	ChrX: 61544–306372^1^	245 Kb	247 Kb	No	***PLCXD1, GTPBP6,*** * PPP2R3B* 4, *SHOX*	***LINC00685*** *, AL732314.1*
DUP5	ChrX: 382644–542740^1^	160 Kb	168 Kb	No^3^	*PLCXD1, GTPBP6, PPP2R3B, SHOX*	*LINC00685, * ***AL732314.1*** *, RP11–309M23.1*
DUP20	ChrX: 11194597–11796693^1^	602 Kb	608 Kb	No^3^	*MID1, HCCS, ARHGAP6, * ***AMELX, MSL3*** *, FRMPD4, PRPS2, TLR7, TLR8, TMSB4X, FAM9C.*	*RP11–120D5.1, AC002981.1, FRMPD4-AS1, TLR8-AS1, RP11–791M20.1, GS1–600G8.5*
DUP26	ChrX: 37283466–37372045^1^	89 Kb	96 Kb	No^3^	*FAM47C, PRRG1* 4, *TM4SF2* 4, *LANCL3, XK, CYBB, DYNLT3, CXorf27, SYTL5.*	*RNU6–49*
DUP40	ChrX: 80225590–80230870^1^	5.3 Kb	28 Kb	No	*BRWD3, HMGN5, SH3BGRL*	*ACA64, U6, AL357115.1*

1 *Genomic positions are in Hg19.*

2 *Non-allelic Homologous Recombination (NAHR).*

3 *Short regions (<300* *bp) with 80% homology were found, but were not considered sufficient for NAHR.*

4 *Gene crossed the minimum and maximum threshold, and is not fully duplicated.*

### Gene Content of Interest and Search in the Database of Genomic Variants (DGV)

**Figure 1:**
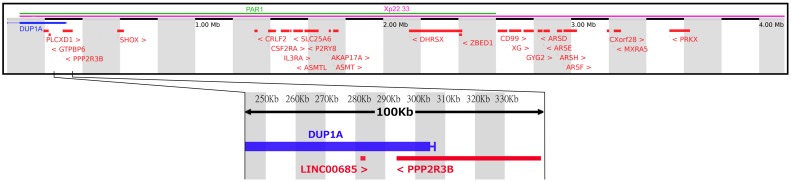
Position of DUP1A relative to *LIN00685* and *PPP2R3B* on the X chromosome (PAR1). Diagram of the Xp22.33, showing the presence of all known protein-coding genes (Red) and DUP1A (Blue). Enlarged is a 100 Kb region showing the location of *PPP2R3B* and *LINC00685* in relation to the DUP1A minimum and maximum. This gain will certainly duplicate the antisense element *LINC00685*, but does not fully duplicate *PPP2R3B* – skewing the ration between the gene and its negative regulator.

DUP1A fully duplicates the following genes: *PLCXD1, GTPB6* and *LINC00685* (Table 3). Apparently, the most interesting is a long non-coding RNA, (*LINC00685*) predicted to act as a negative regulator of a gene (*PPP2R3B*) with a potential role in spermatogenesis (Figure 1). According to the a-CGH data, the *PPP2R3B* gene is affected by DUP1A only for an 11.7 Kb span, thus it is not duplicated but disrupted at intron 7–8 by the CNV (considering the minimum size). Similarly, qPCR data confirm the lack of complete duplication of this gene in all CNV carriers.

Through DGV search within DUP1A, several CNVs (30 duplications and 25 deletions referring to the variant esv27600) were found intersecting the *PLCXD1* and *GTPBP6* loci (located in the proximal part of the duplication), and all of them were identified only in females without further notification on the phenotype. Four variants are reported in close proximity to the most interesting genes, *LINC00685* and *PPP2R3B*. As for *LINC00685,* two distal variants are reported in DGV (4 Kb downstream), esv2219721 and esv266687: the former refers to a 549 bp deletion found in only one man, whereas the latter refers to a 560 bp loss found in 82/1151 (7.1%) subjects (including men and women) analyzed. As for the *PPP2R3B* gene, three variants are reported in DGV and none of them affects the entire gene. The first (esv27923) refers to a 548 bp loss mapping to intron 10–11 of the gene found in one woman over a total of 451 subjects (0.22%); the second (esv25834) refers to a 2.12 Kb region mapping to intron 7–8 of the gene, where both gains (n = 7) and losses (n = 4) were found in 11 women over a total of 451 subjects (2.24%); the third (esv2758560) refers to 3 gains (218.7 Kb) mapping to intron 1–2 of the gene, found in three men over 271 subjects totally analysed (1.1%). All these CNVs seemingly do not disturb the ratio between *LINC00685* and *PPP2R3B* copy number, as with DUP1A.

Within the CNV minimum of DUP5 there is a single predicted microRNA element, *AL732314*.*1* (Table 3). For DUP5, the already described esv2758560 variant is reported in DGV as well as a large number of small CNVs; one variant (nsv508745), describing three insertions (one in a woman and the other two in one man out of 270 belonging to the HapMap project), overlaps with the *AL732314*.*1*. No information about fertility of the single male carrier could be found.

Within the CNV minimum of DUP20, two protein-coding genes are duplicated entirely (Table 3). Of interest is *MSL3*, an homologue of the *Drosophila melanogaster* homonymous gene, which is involved in X-chromosome dosage compensation [16]. A number of CNV annotations can be found in DGV. Of the 3 merged variants (nsv523223: 2.4 Kb; nsv524154: 74.9 Kb; nsv526302: 140.8 Kb) representing 3 gains spanning the *MSL3* locus, no CNVs were found in males. The ten (nsv515163, esv2739963, esv270995, nsv6797, esv271693, nsv510814, nsv6799, esv2739964, nsv436626, nsv499329) common CNVs found upstream of *MSL3* are all found at a much higher frequency in females than in males (28 women (1.66%) *versus* 10 men (0.59%) over a total of 1,682 subjects reported in the DGV).

Both DUP26 and DUP40 do not contain any protein-coding genes but of interest is the presence of *FAM47C*, located 200 Kb downstream of DUP26, and *HMGN5*, located 139 Kb upstream of DUP40 (Table 3). Both these genes show testis-specific expression [14]. Concerning DUP26, three variants are reported to overlap with this CNV: esv1007820 (6.9 Kb), describing a gain found in a man without information on semen parameters; esv28598 (6.3 Kb), describing a gain found in a woman over a total of 451 subjects; esv2740093 (13.6 Kb), describing a loss found in one man over 96 subjects totally analyzed. None of these CNVs intersect the *FAM47C* directly but are located between 307.7–325.6 Kb downstream. Two losses (esv23127, size: 380.5 Kb; nsv510839, size: 74.4 Kb) and 1 gain (esv24190, size: 18.1 Kb) covered the entire DUP40 CNV minimum, whereas one gain (nsv436916, size: 10.26 Kb) covered DUP40 for most of its size (3.8 Kb). Again, these CNVs do not intersect *HMGN5* directly but are located between 238.3–281.1 Kb upstream. The 2 gains and the 2 losses were found at the same frequency in 2 women and 2 men over a total of 508 analyzed subjects (0.39%). No information on the men’s fertility status was available.

## Discussion

A recent study by Tüttelmann et al [4] provides the first statistically significant duplication burden on the X chromosome, reporting a significantly higher number of gains in azoospermic patients compared to both oligozoospermic patients and normal controls. Our analysis of five X-linked CNVs also reveals a significantly higher duplication load in infertile compared to normozoospermic men.

While four of the five CNVs (DUP5, DUP20, DUP26 and DUP40) studied did not individually reach statistical significance, they remained patient-specific. It is worth noting that rare variants have previously been predicted to play an important role in spermatogenic failure [5] due to the strong selection against highly-penetrant infertility-causing variants. All CNVs include, or are in close proximity to, genes with testis-specific expression and potential implication in spermatogenesis. DUP20 contains the *MSL3* gene, a homologue of *Drosophila melanogaster* homonymous gene. *Msl3* in *D. melanogaster* plays a critical role in the X-chromosome dosage-compensation pathway by directing histone H4 acetylation at lysine 16 (H4K16) and the human homologue is thought to have a similar function [17]. Although no information in humans is available, mice models provide evidence that this specific chromatin modification is dramatically increased in elongating spermatids and precedes histone replacement during spermatogenesis, as an initial step of nucleosome removal [18]. Both DUP26 and DUP40 are within close proximity of genes expressed exclusively in the testis. Inside and nearby DUP40 there is a dense area with epigenetic features indicating that DUP40 may disrupt the epigenetic regulation of neighbouring genes. DUP5 is of particular interest due to its large size and location on the PAR1 as explained below.

**Figure 2:**
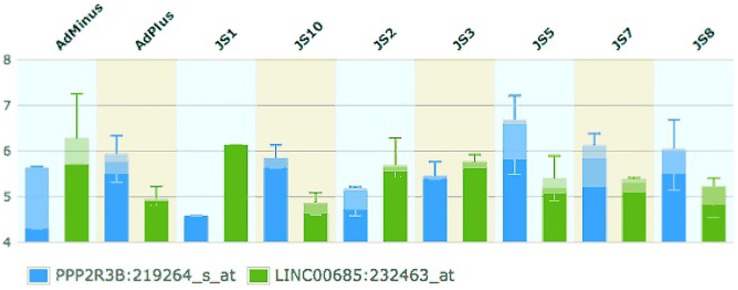
Relative expression of *PPP2R3B* and *LINC00685* in testes biopsies in patients with different phenotypes (Johnsen Score) (Chalmel, et al., 2012). Taken from EBI Expression Atlas. (http://www.ebi.ac.uk/gxa/home).

DUP1A was found at a significantly higher frequency in patients. This gain contains a long non-coding RNA (*LINC00685*) that potentially acts as a negative regulator of a gene with potential role in spermatogenesis, *PPP2R3B*. This proposed mechanism could not be confirmed by functional studies because *in vitro* human spermatogenic cell culture is not available and this antisense is not present in easily accessible model organisms such as mouse and Drosophila. However, expression data provides evidence for an inverse relationship between *PPP2R3B* and *LINC00685* levels in a number of different tissues (see Figure S1 in File S1 and Figure 2) [13], [14]. Concerning the testis, those samples with a high expression of *PPP2R3B* show comparatively low expression of *LINC00685* [13], [14].

Figure 2 shows the changing levels of *PPP2R3B* and *LINC00685* *mRNA* throughout spermatogenesis and the inverse relationship between *PPP2R3B* and *LINC00685*. In tissues that lack germ cells, *PPP2R3B* levels are low, and *LINC00685* levels are comparatively high (AdMinus, JS1 and JS2). *PPP2R3B* levels rise with the presence of mitotically active cells (AdPlus, JS3), and are highest in samples enriched in meiotically active cells (JS5). Scoring system for testicular biopsies was obtained from Chalmel et al [14] and is reported in Table S3 in File S1. Again, in these tissues *LINC00685* levels are decreased. Based on this observation, we propose that the mechanism by which DUP1A could lead to spermatogenic failure is through increased negative regulation, caused by the duplicated *LINC00685* that would decrease *PPP2R3B* transcription in the developing germ cells. This hypothesis is also supported by our qPCR analysis, which proved that the six patients with DUP1A, thus carrying an over-dosage of the antisense *LINC00685*, do not display a duplication of the entire *PPP2R3B* gene.

Although the role of *PPP2R3B* in spermatogenesis has not been explored, indirect functional evidence supports its involvement both in mitosis and meiosis. *PPP2R3B* encodes for a subunit of the protein phosphatase 2 (PP2) protein complex. PP2 is one of four major Ser/Thr phosphatases and has been implicated in a wide range of cellular activities. PP2 is a heterotrimeric protein, composed of a structural A-subunit, a catalytic C-subunit and a regulatory B-subunit. PPP2R3B belongs to this final group, giving specificity to the PP2 complex [19]. In mice, PPP2R3*B*–PP2 was demonstrated to maintain a pool of dephosphorylated CDC6 - a replication-licensing factor [19]. CDC6 phosphorylation and dephosphorylation is necessary for the mitotic G_1_ to S-phase transition [20]. Accordingly, overexpression of *PPP2R3B* results in mitotic arrest at G_1_ phase [19]. Unlike somatic cells, following mitosis primary spermatocytes retain high levels of *CDC6* [20], indicating a possible further role for this protein in meiosis. Expression analysis of testis biopsies with different histology patterns supports the involvement of *PPP2R3B* in mitosis where *PPP2R3B* levels are higher in samples containing mitotically active cells compared to those lacking germ cells. More importantly, the most pronounced expression is observed in biopsies with spermatocytic arrest i.e. those enriched in meiotic cells, indicating an additional role for this gene in meiosis [14]. As mentioned above, *LINC00685* levels show an opposite trend, strongly suggesting a functional link between the two gene products [13], [14] (see supplementary discussion S2, Table S3 and Figure S1 in File S1).

Given that genes that have been recently incorporated into the human X chromosome are involved in male reproductive fitness [12] it is worth noting that *PPP2R3B* has been recently acquired on this chromosome.

Although no CNVs were found in the database of genomic variants (DGV) intersecting *PPP2R3B* or *LINC00685*, re-analysis of raw data deposited in dbVar by Tüttelmann et al. [4] shows that a number of CNVs (5 gains and 2 losses) were found in the PAR1 of azoospermic men. Focusing on the gains, the variant nsv869733 (243.8 Kb), describing 3 gains mapping to the *PPP2R3B* locus, was found exclusively in azoospermic patients (n = 4). The CNV minimums of two of these gains (one mapping to chrX: 298, 292–322, 672 and the other mapping to chrX: 298, 292–330, 801) begin in *PPP2R3B* intron 1–2 and end in intron 12–13, respectively. These CNVs are likely to lead to a non-functional protein by causing an internal duplication. The other CNV (mapping to chrX: 291, 285–336, 040) begins 3.4 Kb upstream of the *PPP2R3B* locus and ends in intron 12–13, which would disrupt the gene at exon 1 as well as the promoter. Moreover, the presence of a predicted promoter 1Kb upstream of *PPP2R3B* and several regions enriched in histone methylation suggests that these gains may disrupt the correct regulation of *PPP2R3B*. The duplication of the *PPP2R3B* locus has been reported also in men with abnormal karyotype and AZF deletions and in a single man with normal karyotype and AZFb deletion [21]. However, in these cases the infertile phenotype is clearly related to the karyotype and Y chromosome defects. The observations from Tüttelmann, together with ours, showing the relatively high frequency at which DUP1A was found exclusively in patients and its potential link to *PPP2R3B* gene expression, strongly indicate that *PPP2R3B* could be considered a novel spermatogenesis candidate gene.

Large CNVs of the PAR1 region, like DUP1A and DUP5, may lead to impaired spermatogenesis also through a structural effect disturbing male meiosis i.e. altering the recombination event that occurs between the PAR1 regions of the sex chromosomes [8], [9]. PAR1 recombination becomes progressively more frequent towards the distal telomeric boundary [22], where DUP1A and DUP5 are located, showing the importance of this region during meiosis. For instance, the merged analysis of data by Tüttelmann and our previous study shows that no PAR-linked CNVs were found in the vicinity of these 2 CNVs in normozoospermic controls, with the exception of one subject found by Krausz et al. to be carrying DUP4A. However, this CNV only partially overlaps with the DUP1A (at its distal boundary) and DUP5 (at its proximal boundary). This observation may indicate that: i) the pathogenic effect of DUP1A is more likely related to the misbalanced *LINC00685/PPP2R3B* gene dosage effect; ii) the portions of DUP1A and DUP5 that are not overlapping with DUP4A could be affecting specific sites of importance for X-Y pairing during meiosis.

Finally, the combination of all data discussed above supports the importance of the PAR1–linked CNVs in male infertility. Our most relevant finding is the identification of the first recurrent, X-linked gain associated with spermatogenic failure, DUP1A. Two possible mechanisms have been provided to explain the pathogenesis of the associated infertile phenotype – one identifying and regarding a novel spermatogenesis candidate gene and another due to a potential structural effect in the PAR1. Both of these scenarios are intriguing and prompt further research.

## Supporting Information

File S1 **Materials and Methods:** S1**. Table S1.** Impact of CNVs on Total Sperm Count (TSC) and Total Motile Sperm Count (MSC) Comparison was performed between carriers and non-carriers, excluding controls (A) and including controls (B). **Table S2.** Primers used for qPCR Analysis. **Discussion** **:** S2. **Table S3** - Scoring system for testicular biopsies (Johnsen score). **Figure S1.** Relative expression of PPP2R3B and LINC00685 in Human organs taken from EBI Expression Atlas.
(DOC)
