# Supplementary material for: X Chromosome-Linked CNVs in Male Infertility: Discovery of Overall Duplication Load and Recurrent, Patient-Specific Gains with Potential Clinical Relevance
Source: PLoS One. 2014 Jun 10;9(6):e97746. doi: 10.1371/journal.pone.0097746 (PMC4051606; doi:10.1371/journal.pone.0097746)
Supplement: File S1 — Materials and Methods: S1. Table S1. Impact of CNVs on Total Sperm Count (TSC) and Total Motile Sperm Count (MSC) Comparison was performed between carriers and non-carriers, excluding controls (A) and including controls (B). Table S2. Primers used for qPCR Analysis. Discussion : S2. Table S3 - Scoring system for testicular biopsies (Johnsen score). Figure S1. Relative expression of PPP2R3B and LINC00685 in Human organs taken from EBI Expression Atlas. (DOC) [file pone.0097746.s001.doc]

**FILE S1: Combined Supporting Information files**

**Supplementary Materials and Methods S1**

**Patient samples used in the study had an average sperm concentration of 1.3 ± 2.6 x10^6^ spermatozoa.mL^-1^, an average total sperm count of 2.6 ± 7.3 x10^6^, and an average total motile sperm count of 1.4 ± 2.7 x10^6^. Control samples used in the study had an average sperm concentration of 98 ± 70 x10^6^ spermatozoa.mL^-1^, an average total sperm count of 298 ± 207 x10^6^, and an average total motile sperm count of 197 ± 136 x10^6^.**

| **Table S1.** Impact of CNVs on Total Sperm Count (TSC) and Total Motile Sperm Count (MSC) Comparison was performed between carriers and non-carriers, excluding controls (A) and including controls (B). | | | | | |
| --- | --- | --- | --- | --- | --- |
| **A.** | | | | | |
| **CNV** | **Type** | **Mean**  **TSC** | **Mean**  **TMSC** | **p-value (TSC)** | **p-value (TMSC)** |
| DUP1A | Carriers (n = 6) | 3.3 ± 3.6 | 0.5 ± 0.4 | *p = 0.21* | *p = 0.22* |
|  | Non-Carriers (n = 428) | 2.6 ± 7.3 | 1.5 ± 2.7 |  |  |
| DUP5 | Carriers (n = 2) | 4.3 ± 6.0 | 0.5 ± 0.6 | *p = 0.88* | *p = 0.3* |
|  | Non-Carriers (n = 274) | 2.6 ± 7.3 | 1.4 ± 2.7 |  |  |
| DUP20 | Carriers (n = 1) | 7.5 | 3.6 | *p = 0.11* | *p = 0.12* |
|  | Non-Carriers (n = 275) | 2.6 ± 7.3 | 1.4 ± 2.7 |  |  |
| DUP26 | Carriers (n = 1) | 0.0 | 0.0 | *p = 0.22* | ***p = 0.09*** |
|  | Non-Carriers (n = 275) | 2.6 ± 7.3 | 1.4 ± 2.7 |  |  |
| DUP40 | Carriers (n = 3) | 0.1 ± 0.1 | 0.0 | *p = 0.15* | ***p = 0.004*** |
|  | Non-Carriers (n = 273) | 2.6 ± 7.4 | 1.4 ± 2.7 |  |  |
| **B.** | | | | | |
| **CNV** | **Type** | **Mean**  **TSC** | **Mean**  **TMSC** | **p-value (TSC)** | **p-value (TMSC)** |
| DUP1A | Carriers (n = 6) | 3.3 ± 3.6 | 0.5 ± 0.4 | *p = 0.35* | ***p = 0.008*** |
|  | Non-Carriers (n = 935) | 156 ± 195 | 136 ± 139 |  |  |
| DUP5 | Carriers (n = 2) | 4.3 ± 6.0 | 0.5 ± 0.6 | ***p =*** *0.29* | *p =0.051* |
|  | Non-Carriers (n = 601) | 144 ± 205 | 135 ± 145 |  |  |
| DUP20 | Carriers (n = 1) | 7.5 | 3.6 | *p = 0.99* | *p = 0.089* |
|  | Non-Carriers (n = 602) | 144 ± 205 | 135 ± 145 |  |  |
| DUP26 | Carriers (n = 1) | 0.0 | 0.0 | *p = 0.15* | ***p = 0.003*** |
|  | Non-Carriers (n = 602) | 144 ± 205 | 135 ± 145 |  |  |
| DUP40 | Carriers (n = 3) | 0.1 ± 0.1 | 0.0 | ***p = 0.03*** | ***p = 0.03*** |
|  | Non-carriers (n = 600) | 144 ± 205 | 135 ± 145 |  |  |

| **Table S2.** Primers used for qPCR Analysis | | | | |
| --- | --- | --- | --- | --- |
| **CNV** | **Sequence** | **Length** | **Loci (Hg19)** | **Product Length** |
| DUP1A | FOR: CGGGGCTCGTCTTTCTACG | 19 | ChrX:231,729-231,747 | 91bp |
|  | REV: GGTGGCTACATTTCCGCAC | 19 | ChrX:231,818-231,799 |  |
| DUP5 | FOR: CCCGGATTTGGGGTTGACAG | 20 | ChrX:406,639-406,659 | 114bp |
|  | REV: CTGACTCCCCCAGCTACTCC | 20 | ChrX:406,752-406,732 |  |
| DUP20 | FOR: GCAGCCTCATCACCACATCC | 20 | ChrX:11,316,760-11316780 | 90bp |
|  | REV: ATGGAGTGTTGGCCAGGAAC | 20 | ChrX:11,316,849-11,316,829 |  |
| DUP26 | FOR: GACCACACCGCTTTACCCTG | 20 | ChrX:37,364,527-37,364,547 | 97bp |
|  | REV: CCTCGCAGCTGAGATGGTTG | 20 | ChrX:37,364,623-37,364,603 |  |
| DUP40 | FOR: GCATGCTCCCCAATGTAGGTC | 21 | ChrX:80,229,910-80,229,930 | 90bp |
|  | REV: GATGCTAAACGTCTGCTTTGACTG | 24 | ChrX:80,229,999-80,229,979 |  |
| *PPP2R3B* | FOR: AGCGTCTGGCTGTCATTCAA | 20 | ChrX:343,567-343,586 | 121bp |
|  | REV: GGCCAGGACTCAAGAAGCAT | 21 | ChrX:343,668-343,687 |  |
| *PMP22* | FOR: CCTTCTCAGCGGTGTCATC | 19 | ChrX:15,134,216-15,134,235 | 64bp |
|  | REV: ACAGACCGTCTGGGCG | 17 | ChrX: 15,134,262-15,134,279 |  |

**Supplementary Discussion S2. Expressional Analysis of PPP2R3B and LINC00685**

Data by Chalmel et al (14) shows the changing levels of PPP2R3B and LINC00685 throughout spermatogenesis and the inverse relationship between PPP2R3B and LINC00685. Scoring system is reported in Table S3. In tissues which lack germ cells, PPP2R3B levels are low, and LINC00685 levels are comparitively high (AdMinus, JS1 and JS2). PPP2R3B levels rise with the presence of mitotically-active cells (AdPlus, JS3), and are highest in samples enriched in meiotically active cells (JS5). Again, in these tissues LINC00685 levels are decreased.

**Table S3 - Scoring system for testicular biopsies (Johnsen score).**

| **Score** | **Histological criteria** |
| --- | --- |
| 10 | Full spermatogenesis |
| 8 | Less than five spermatozoa per tubule, few late spermatids |
| 7 | No spermatozoa, no late spermatids, many early spermatids |
| 5 | No spermatozoa or spermatids, many spermatocytes |
| 3 | Spermatogonia only |
| 2 | No germinal cells, Sertoli cells only |
| 1 | No seminiferous epithelium |
| AdPlus | Presence of Ad spermatogonia (Adolescent Cryptorchidism) |
| AdMinus | Absence of Ad spermatogonia (Adolescent Cryptorchidism) |

**Supplementary Figure S1 -** Relative expression of PPP2R3B and LINC00685 in Human organs taken from EBI Expression Atlas. The levels of PPP2R3B and LINC00685 show an inverse relationship, i.e. tissues with a high levels of PPP2R3B tend to show a low level of LINC00685 expression.

**
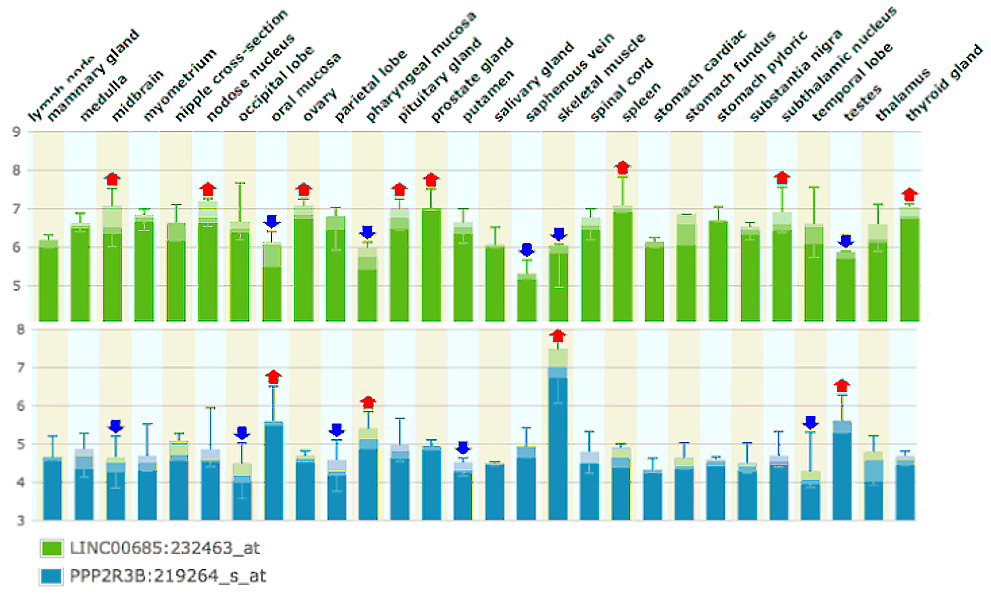
**
